# The Efficacy of Inhaled Phage Cocktail to Prevent Ventilator-Associated Pneumonia in Children: A Double-Blind Clinical Trial

**DOI:** 10.3390/biomedicines13092103

**Published:** 2025-08-29

**Authors:** Mohammad Reza Navaeifar, Golnar Rahimzadeh, Raha Rezai, Hormoz Ranjbar, Kofi Asare-Addo, Ali Nokhodchi, Mohammad Sadegh Rezai

**Affiliations:** 1 Pediatric Infectious Diseases Research Center, Communicable Diseases Institute, Mazandaran University of Medical Sciences, Sari 4815733971, Iran; navaifar@gmail.com (M.R.N.); rahimzadehgolnar@yahoo.com (G.R.); rezai@mazums.ac.ir (R.R.); hormozranjbar2014@gmail.com (H.R.); 2 Department of Pharmacy, University of Huddersfield, Huddersfield HD1 3DH, UK; k.asare-addo@hud.ac.uk; 3 Pharmaceutics Research Lab, School of Life Sciences, University of Sussex, Brighton BN1 9QJ, UK

**Keywords:** phage cocktail, inhalation, ventilator-associated pneumonia (VAP), children

## Abstract

**Background/Objectives**: Phage therapy is gaining attention as a potential alternative to antibiotics. This study investigates the potential use of a phage cocktail as a preventive measure against ventilator-associated pneumonia (VAP) in children. **Methods**: Sixty patients were selected from the pediatric intensive care units for this double-blind clinical trial. The inclusion criteria involved patients requiring invasive mechanical ventilation for more than two days. This, however, excluded newborns and children with bacterial pneumonia. The intervention group received the standard country protocol drugs in addition to 5 mL of phage cocktail suspension administered every 24 h through an in-line mesh nebulizer for 7 days. The two groups were compared in terms of the incidence of VAP, survival rate, and duration of hospitalization. **Results**: The intervention with phages had a significant impact on reducing the occurrence of VAP compared to the group receiving a placebo. The data showed that there was a significant difference in the occurrence of VAP between the two groups, with a lower percentage of VAP in the phage cocktail group (*p* < 0.05). Additionally, the cultures of specific bacterial strains did not yield positive results. Notably, there were no significant differences between the intervention and placebo groups in terms of mortality rates and duration of hospitalization (*p* > 0.05). **Conclusions**: Inhalation of a phage cocktail shows a promising effect in preventing VAP in PICU patients at a tertiary hospital in Iran with no observed side effects. However, further, larger clinical trials are necessary to validate its efficacy.

## 1. Introduction

Ventilator-associated pneumonia (VAP) is the second most common nosocomial infection in the pediatric intensive care unit (PICU) after bloodstream infections. It accounts for up to 20% of all such infections. VAP can lead to increased costs, prolonged hospital stays, morbidity, and mortality [1]. The range of reported incidence rates of VAP is typically between 1.4 and 7 episodes per 1000 ventilator days in developed countries [1,2]. The higher rates of respiratory infections in developing countries range from 16.1 to 89 episodes per 1000 ventilator days. This discrepancy may be due to various factors, including differences in healthcare infrastructure, infection control practices, and antibiotic resistance patterns.

VAP can be classified based on the time of its occurrence into early-onset and late-onset pneumonia. Early-onset pneumonia is usually caused by bacteria such as Staphylococcus aureus, Haemophilus influenza, and Streptococcus pneumoniae. In contrast, late-onset pneumonia is commonly associated with more resistant bacteria, such as methicillin-resistant Staphylococcus aureus (MRSA), Pseudomonas aeruginosa, or Enterobacter species. Understanding the differences in causative pathogens between early-onset and late-onset VAP is crucial for appropriate diagnosis and treatment selection [3,4,5].

The overuse of antibiotics in treating lung infections has led to the emergence of multidrug-resistant (MDR) bacteria and strains that produce extended-spectrum β-lactamases (ESBLs). These bacteria have become resistant to multiple antibiotics due to genetic changes and the production of enzymes that can break down the antibiotics’ structure. As a result, the treatment of respiratory infections has become more expensive and difficult, thus leading to increased mortality rates. There is therefore a pressing need for an effective solution in preventing and treating these infections [6,7,8].

Bacteriophages have gained attention as a potential treatment for infections [9,10,11]. In this context, exploring alternative therapies such as phage cocktails, which specifically target and kill bacteria, including antibiotic-resistant strains, could offer a promising solution. Phages can be tailored to target specific bacterial strains, thus providing a targeted approach to treating infections while potentially reducing the risk of resistance development. In a bid to reduce the possibility of phage resistance, a phage cocktail containing two or more phages is recommended in phage therapy [9,10,11]. Over the past five years, there have been 20 confirmed instances of a phage cocktail being used to treat respiratory infections. The availability of therapeutic phage preparations for clinical use in countries like Georgia and Russia reflects the growing interest and acceptance of phage therapy as a treatment option for bacterial infections. Commercial phages that target bacteria commonly associated with lung infections, such as Pseudomonas aeruginosa and Staphylococcus aureus, are being produced by Microgen in Russia. This development represents a significant step forward in the production and availability of phage-based treatments for respiratory infections caused by these pathogens.

The use of phage cocktails in respiratory infections presents a promising alternative or a complementary option to antibiotics [11,12,13,14]. Phage therapy can also be considered for patients who are intolerant to antibiotics due to allergies or severe complications. There is a growing interest in using phage cocktails for the prevention or eradication of MDR infections. The effectiveness, however, of its prophylactic use for respiratory infections is currently unknown [12]. Also, the socio-economic cost of treating VAP is significantly higher than the cost of preventing it. Hence, it is highly crucial to adopt preventive measures to minimize the risk of VAP. This study, therefore, investigates the efficacy of inhaled phage cocktails in preventing VAP among children admitted to the PICU at a tertiary hospital in Sari, Iran. In other words, this clinical study corresponds to a Phase II randomized, double-blind, placebo-controlled trial, focusing on the evaluation of the efficacy and preliminary safety of an inhaled phage cocktail in preventing ventilator-associated pneumonia (VAP) in pediatric patients. This study builds upon initial safety and feasibility work, aiming to determine the effectiveness of the intervention in a controlled clinical environment [12].

## 2. Materials and Methods

### 2.1. Host Cell

The phage cocktail used in this clinical trial was specially formulated to target three major bacterial pathogens commonly linked to VAP in PICUs. These strains were chosen based on prior microbiological surveillance data from our hospital’s PICU, which indicated frequent isolation of these pathogens in VAP cases. The targeted bacteria include *Pseudomonas aeruginosa* (ATCC No. 27853), *Acinetobacter baumannii* (ATCC No. BAA-1605), and Methicillin-resistant *Staphylococcus aureus* (ATCC No. 33591), which were purchased for testing from the Pasteur Institute, Tehran, Iran.

### 2.2. Isolation of Bacteriophages

A 1000 mL sewage sample was collected from a tertiary hospital in Sari, Iran. The sample was mixed with Luria Broth (LB) (QUELAB, Albuquerque, NM, USA) (200 mL) and host cells, incubated for 24 h (37 °C, 150 rpm), and centrifuged (7000× *g*, 15 min). The resulting supernatants were filtered using a 0.22 μm filter under sterile conditions. It is worth noting that the lytic phages against *Pseudomonas aeruginosa* and *Acinetobacter baumannii* were obtained as reported in our previous study [12].

### 2.3. Characterization of Phage Cocktail

To determine the phage titer, the Double-Layer Agar (DAL assay) test was used. The phage cocktail was serially diluted in 12 sterile tubes, and positive and negative controls were included. The diluted phage cocktails were mixed with bacteria and transferred to the top agar. After incubation (37 °C, 24 h), the phage titer was determined by counting the plaques formed on the plates [12].

Plaque-forming Units (PFU)/mL = (Number of plaques counted) × 10 × (1/Dilution factor)

To observe the phage cocktail using Transmission Electron Microscopy (TEM), the supernatant was centrifuged (7000× *g*, 60 min) and placed onto a carbon-coated copper grid. After staining with 2% uranyl acetate, the phage cocktail was imaged using the advanced Zeiss EM 900 TEM (Carl Zeiss AG, Oberkochen, Germany) at 120 kV [12].

### 2.4. Detoxification of Phage Cocktail

The phage cocktail was mixed with Triton X-100 (3% *v*/*v*) and incubated at 25 °C. Activated carbon (20%) was added, and the solution was centrifuged (5000× *g*, 10 min). The supernatant was filtered using a 0.45 µm filter membrane, and phage titer and viability were determined via the previously defined DLA assay [12].

### 2.5. Limulus Amebocyte Lysate (LAL) Test

The LAL test (ENDOSAFE, Charleston, SC, USA) was conducted to assess the detoxification of the phage cocktail supernatant. The test has a sensitivity of 0.06 EU/mL and an endotoxin limit of 5 EU/mL. *Escherichia coli* 055: B5 was employed as a positive control [12].

### 2.6. The Clinical Phase

This study was conducted in the pediatric intensive care unit (PICU) of a tertiary hospital in Sari, Iran, from August 2023 to December 2023. It is a Phase II randomized, double-blind, placebo-controlled trial that received approval from the Ethics Committee at Mazandaran University of Medical Sciences (IRB number IR.MAZUMS.REC.1400.305) on 23 June 2021. This study has been registered at www.clinicaltrials.gov with the code number IRCT20230701058628N1.

**Figure 1:**
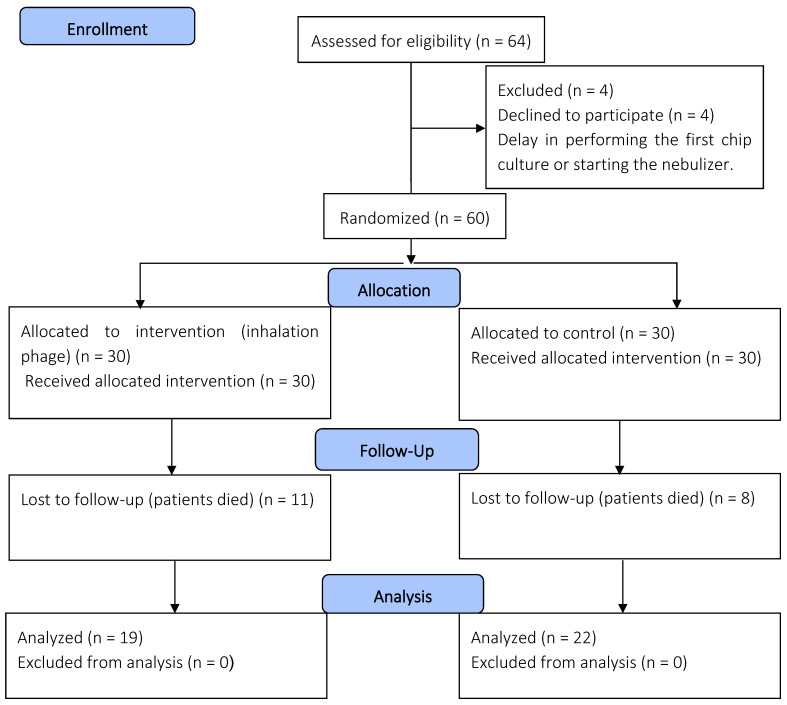
A depiction of the flowchart used for the clinical phase.

All patients who participated in this study provided their parents’ or legal guardians’ consent and agreement beforehand. This study involved patients under 18 years of age who were admitted to the PICU. The inclusion criteria involved patients requiring invasive mechanical ventilation for more than two days; however, newborns and those with bacterial pneumonia were excluded (Figure 1). The decision to confirm the diagnosis of primary bacterial pneumonia was based on a combination of clinical, laboratory, and radiological evidence [3,4]. In both study groups, a phage combination or placebo was administered to the patients from the first day of mechanical ventilation until 7 days.

### 2.7. Intervention

In this study, participants who met the criteria were randomly assigned to one of two groups using randomization software (Random Allocation Software, version 1.0): the phage cocktail group and the placebo group. Each group consisted of 30 patients, and it is important to note that the researchers were not involved in selecting patients for either group. The phage and placebo vials were designed to look identical, with each vial assigned a specific code.

Participants in the phage cocktail group received standard country protocol medications along with a 5 mL dose of phage cocktail suspension (10^12^ PFU/mL) every 24 h, administered via an inline mesh nebulizer for 7 days. In contrast, the placebo group received the standard medications along with a 5 mL suspension without phage, also every 24 h using a nebulizer for the same duration.

Since the intervention targeted the tracheobronchial tree rather than the lung parenchyma, there were no specific ventilator settings required during nebulization. The nebulizer was positioned in the inspiratory limb of the ventilator circuit, approximately 15 cm from the endotracheal tube, to ensure optimal delivery of the phage cocktail to the airways.

### 2.8. Primary and Secondary Outcome Assessment

The patient’s health was monitored daily from the start of treatment until their hospital stay ended. Cultivation was carried out using the non-bronchoscopic bronchoalveolar lavage (NB-BAL) method before intervention. This was performed at baseline and then every three days, resulting in three samples during the 7-day intervention (days 0, 3, and 6). The criteria for diagnosing ventilator-induced respiratory infections were based on the National Healthcare Safety Network (NHSN) guidelines. If the results of a culture test were positive, the pathogen type was identified using conventional microbial methods, and the antibiotic sensitivity pattern was analyzed using the Kirby–Bauer method. The primary outcome of this monitoring was to detect a positive tracheal culture or the occurrence of VAP, while the secondary outcome was to track the patient’s status, discharge from the PICU, discharge from the hospital, or death.

Although this study focused on prevention rather than treatment, clinical assessment for signs and symptoms of VAP was performed daily. Clinical criteria for diagnosing VAP were based on the National Healthcare Safety Network (NHSN) guidelines, which include a combination of new or progressive infiltrates on chest X-ray, fever or hypothermia, leukocytosis or leukopenia, purulent tracheal secretions, deterioration of oxygenation (decreased PaO2/FiO2 ratio), and changes in respiratory status such as increased ventilator settings; a patient was considered to have a clinical cure if these signs were absent or resolved without evidence of VAP during the observation period. In our clinical trial, patients who received the inhaled phage cocktail were closely monitored for adverse events, including acute respiratory distress syndrome (ARDS), cytokine storms, allergic reactions, and endotoxin-related reactions.

### 2.9. Sample Size Calculation and Statistical Analysis

Data analysis was performed using SPSS software version 24. The Kolmogorov–Smirnov test was employed to check data normality. Non-parametric Wilcoxon equivalent tests and variance analysis were used for abnormal data. For qualitative variables, the chi-square test was used. A *p* < 0.05 was considered significant. The sample size was calculated according to the following formula:

$$N=\frac{2\sigma^{2}(Z_{1-\alpha /2}+Z_{1-\beta }{)}^{2}}{\Delta^{2}}$$

N =60;
σ^2^ = variance of the outcome;
Δ = expected difference between the two groups;
Z_1−α/2_ = Z-value corresponding to the significance level (α = 0.05 → Z ≈ 1.96);
Z_1−β_ = Z-value corresponding to the study power (80% power → Z ≈ 0.84).

## 3. Results

### 3.1. Characterization of Phage Cocktail

**Figure 2:**
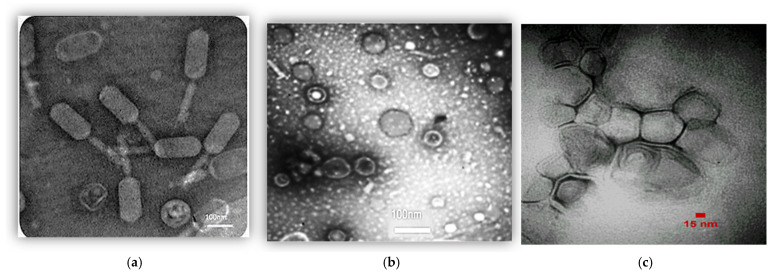
Phages belonging to (**a**) *Siphoviridae* family, (**b**) *Cystoviridae* family, and (**c**) *Podoviridae* family.

The concentration of the phage cocktail was determined to be 10^12^ PFU/mL. The phenotype of the phage mixture was observed using TEM (Figure 2). It appears that the phage cocktail consists of three distinct types of phages characterized by their unique morphological characteristics: *Siphoviridae* (Figure 2a), *Cystoviridae* (Figure 2b), and *Podoviridae* (Figure 2c).

### 3.2. Detoxification of Phage Cocktail

The phage cocktail detoxification test showed that the cocktail (0.06 EU/mL and 5 EU/mL of endotoxin) did not clot after 1 h at 37 °C. The positive control clotted. The phage titer remained unchanged and effective after detoxification.

### 3.3. Participants in the Clinical Phase

Between August and December 2023, Boo Ali Sina Hospital’s PICU monitored 60 patients, randomly divided into two groups of 30 each: phage cocktail and placebo. Four patients were excluded due to a delayed start of treatment. Table 1 provides an overview of patient characteristics such as age, sex, and body mass index (BMI). The two groups exhibited a significant difference in BMI (*p* < 0.05), indicating that this factor may be important in the study’s outcomes.

### 3.4. Primary and Secondary Outcome

The results of the present study show that 43.5% of the patients who developed a pulmonary infection during their hospital stay had a history of positive tracheal culture results. A total of 65.2% of these patients had either fever or hypothermia, while 56.5% reported worsening sputum. Only 8.7% experienced cough or respiratory distress. Deterioration of oxygenation was observed in 91.3% of the patients, while leukocytosis was reported in 39.1% and leukopenia in 13%. Around 69.6% of the patients showed tachycardia or bradycardia, and 13% had apnea or hemoptysis. New infiltration in the lung X-ray was observed in 60.9% of the patients. Consolidation in the lung X-ray was observed in 3.4% of the patients, but none of them had cavitation. There was also a decrease in the number of positive cultures for *Pseudomonas aeruginosa*, *Acinetobacter baumannii*, and MRSA. However, cultures of patients who acquired lung infections during hospitalization revealed that the most common organisms were Pseudomonas aeruginosa and *Klebsiella pneumoniae* (Table 2). 

The median risk score of PRISM (Pediatric Risk of Mortality) children’s death in the whole study population was 4 (IQR, 2–7), and this value was 5 (IQR, 2–8) and (IQR, 1.7–75) in the phage and placebo groups, respectively. Among the patients, 31.7% (19 patients) died. A total of 26.7% were from the placebo group (8 patients), and 36.7% were from the phage cocktail group (11 patients). However, there was no significant difference observed between the two groups in terms of mortality. Eight deaths were reported due to respiratory issues, out of which two occurred in the placebo group, while six were recorded in the phage cocktail group (*p*-value of 0.254).

The median hospitalization period for the patients was 27 days (IQR, 14–42). In the phage cocktail group, this value was 28 days (IQR, 17–51), while in the placebo group, it was 23 days (IQR, 12–37) (*p*-value of 0.158). The median duration of ventilation of the patients was 12 days (IQR, 8–21), with the value in the phage and placebo groups being 16 (IQR, 9–25) and 12 (IQR, 8–18) days, respectively (*p*-value of 0.310). No cases of acute respiratory distress syndrome (ARDS) or clinical signs indicative of a cytokine storm, such as a sudden deterioration in respiratory function or elevated inflammatory markers, were observed in the group that received phage treatment throughout the study period. Additionally, there were no allergic reactions reported; none of the patients exhibited symptoms such as rash, swelling, itching, hives, or low blood pressure that could be attributed to phage inhalation.

## 4. Discussion

This study sought to evaluate the effectiveness of a phage cocktail in preventing VAP in children at a tertiary hospital in Sari, Iran. This study was performed as a double-blind trial, with the results showing that patients who received the phage cocktail experienced a significantly lower occurrence rate of VAP (23.4%) as compared to those who were given a placebo (53.4%) (*p*-value = 0.033). The most commonly detected pathogen in positive cultures was Klebsiella pneumoniae, with a prevalence rate of 31.1%. VAP is a serious concern for patients in the PICU. The mortality rate of VAP is higher than other forms of hospital-acquired pneumonia, at approximately 71% [1,2,3,4]. The use of antibiotics for treating VAP has also been associated with several issues, including the emergence of MDR pathogens and insufficient efficacy in some cases. As a result, alternative therapeutic approaches such as phage therapy are being considered as a potential solution [12].

Kakasis et al.’s clinical trial showed that phage therapy significantly reduced VAP incidence compared to antibiotics. This suggests that phage therapy may be an effective preventive measure for VAP in this patient population. Our study results support this conclusion [15].

In a case report by Tan et al., it was found that phage was effective in clearing hospital-acquired pneumonia (HAP) caused by carbapenem-resistant Acinetobacter baumannii in an 88-year-old man. This treatment helped in the recovery of lung function and increased the blood oxygen saturation level. Phage therapy was also found to be effective in reducing infection and inflammation in the lung tissue, which in turn improved alveolar oxygen absorption [16]. In 2018, Hoyle et al. published a remarkable case study that revealed the efficacy of phage treatment in a 17-year-old female patient with cystic fibrosis and chronic infection. The patient was treated with phages once daily (3 × 10^8^ PFU/mL) using a compression nebulizer and also orally received phages twice daily for 20 days. Following the initial findings of phage therapy, the patient’s condition significantly improved. Dyspnea was eliminated, and cough frequency decreased. This study provided a promising platform for the use of phage therapy in treating cystic fibrosis and other chronic infections [17].

Based on the present study, *Klebsiella pneumoniae*, Enterobacter, and *Staphylococcus saprophyticus* were found to be the most frequent bacteria in positive cultures. However, *Pseudomonas aeruginosa*, *Acinetobacter baumannii*, and MRSA were not identified in the positive fourth and fifth cultures. The phage cocktail used in this study contained *Siphoviridae*, *Cystoviridae*, and *Podoviridae*. These phages had specifically lytic activity against MRSA, *Pseudomonas aeruginosa*, and *Acinetobacter baumannii*, respectively. This means that the absence of bacteria such as MRSA, *Pseudomonas aeruginosa*, and *Acinetobacter baumannii* can be attributed to the targeted lytic activity of the phages in the phage cocktail. On the other hand, the phage cocktail did not contain phages against *Klebsiella pneumoniae*, Enterobacter, and *Staphylococcus saprophyticus*, making the presence of these bacteria in a positive culture thus expected. The group that received the phage cocktail had a significantly lower infection rate than the group that received a placebo (*p* < 0.05). The phage cocktail can therefore effectively prevent VAP.

In 2023, our research group conducted a study and reported significant positive results for an inhaled phage cocktail. The therapy resulted in a decrease in fever, dyspnea, negative sputum culture, duration of hospitalization, and no secondary infections for both the control and phage-receiving groups [12]. This present study, however, showed there was no significant difference in the mortality rate and the median hospitalization period for the patients between the two groups (*p* > 0.05). This could be attributed to the longer hospitalization period and, therefore, the severity of the underlying disease of the patients. Further research is needed to investigate mortality and other related indicators to confirm this result.

In the current study, the cocktail phage was administered via nebulization. Possible adverse events such as rash, edema, itching, hives, and hypotension were not observed in patients from the inhalation of phage therapy. Wu et al. conducted a study of four critically ill COVID-19 patients with pulmonary carbapenem-resistant Acinetobacter baumannii infections who were treated with ɸAb124 phage. One of the patients experienced an atypical cytokine storm of IL-6 and IL-8 within 4 h of phage administration. The patient’s IL-6 and IL-8 levels also returned to normal within 1 day [11]. Two studies by Debarbieux et al. (2010) [18] and Yajun et al. (2021) [19] demonstrated the effectiveness and safety of phage therapy in mice. In the Debarbieux et al. (2010) study, intranasal administration of phage led to a significant decrease in the levels of pro-inflammatory cytokines IL-6 and TNF-α in the mouse lungs [18]. Similarly, in the Yajun et al. (2021) study, it was observed that the intratracheal aerosol delivery of phages did not cause any adverse effects in mice exposed to *Pseudomonas aeruginosa* through a liquid aerosol. These findings highlight the potential usefulness of phage therapy as a safe and effective treatment option for lung infections [19].

A mesh nebulizer is an effective method to deliver phage particles to the lungs via inhalation. According to Chang et al. (2018), nebulization is more efficient than intranasal instillation in transporting phage particles to the lungs [20]. The group of mice that received phage through intraperitoneal injection (i.p.) only showed a bacterial load reduction of 0.5 log. A significant 2-log reduction was observed in the group treated through inhalation.

This study has several limitations that should be acknowledged. Immune markers and antibody titers were not directly measured; however, their assessment is recommended for future research. Additionally, this study did not address quantitative bacteriology, bacteriophage load, or inflammatory factors in the lungs. Furthermore, this study was conducted solely in the northern region of Iran, which limited its sample size and may have affected the specificity of the clinical criteria, potentially leading to an over- or underestimation of the prevalence of ventilator-associated pneumonia (VAP) in the groups. Future research should involve multiple regions to allow for more generalized conclusions.

## 5. Conclusions

In conclusion, this study demonstrated that patients who received the inhaled phage cocktail had a significantly lower rate of ventilator-associated pneumonia (VAP) compared to those who were given a placebo. No side effects were reported when the phage cocktail was inhaled. While these results are promising, the authors emphasize the need for further extensive research involving larger patient groups from various regions of the country to confirm the effectiveness of inhalation phage therapy for treating pneumonia. Therefore, caution should be exercised when generalizing the reported findings.

## Figures and Tables

**Table 1:** Data of patients in two groups.

		Phage Cocktail (n = 30)	Placebo (n = 30)	Total	*p*-Value
Gender (%)	Female	46.7%	43.3%	45%	0.795 **
	Male	53.3%	56.7%	55%	
Age (month) mean ± SD		72.68 ± 47.65	63.55 ± 80.10		0.592 *
BMI z-score, mean ± SD		1.05 ± 0.15	0.95 ± 0.35		0.051 *
Contracting COVID-19	Yes	20%	13.3%	16.7%	0.488 **
	No	80%	86.7%	83.3%	
Cuffed endotracheal tubes	Yes	63.3%	53.3%	58.3%	0.601 **
	No	36.7%	46.7%	41.7%	
Previous positive blood culture history	Yes	55.6%	41.7%	47.6%	0.670 **
	No	44.4%	58.3%	52.4%	
Medical history	Respiratory	26.7%	23.3%	25%	
	Non-respiratory infectious	13.3%	13.3%	13.3%	
	Gastrointestinal–hepatic	3.3%	-	1.7%	
	Cerebral–nervous	33.3%	-	35%	
	Metabolic glands	-	3.3%	1.7%	
	Blood-cancer	6.7%	3.3%	5%	
	Allergy–immunodeficiency	3.3%	-	1.7%	
	Surgery–trauma	13.3%	10%	11.7%	
	Burn	-	6.7%	3.3%	
	Other	-	3.3%	1.7%	

Analysis of variance test *, Chi-square test **.

**Table 2:** Reporting primary and secondary outcomes.

Outcome	Phage Cocktail (n = 30)		Placebo (n = 30)		*p*-Value			
NO VAP	76.60%		46.60%		0.033 **			
Survival rate	63.30%		73.30%		0.580 **			
Frequency of positive cultures (%)								
Microorganisms/days	*P. aeruginosa*		*A. baumannii*		MRSA *		*K. pneumonia*	
First culture	placebo	phage cocktail	placebo	phage cocktail	-		placebo	phage cocktail
	20%	0	1.7%	0			6.3%	2%
Second culture	placebo	phage cocktail	placebo	phage cocktail	placebo	phage cocktail	placebo	phage cocktail
	25%	0	6.7%	0	1.7%	0	9.7%	4%
Third culture	placebo	phage cocktail	placebo	phage cocktail	-		placebo	phage cocktail
	13.3%	0	6.7%	0			10%	3.3%
Fourth culture	-		-		-		placebo	phage cocktail
							1.3%	2%
Fifth culture	-		-		-		placebo	phage cocktail
							1.7%	0

* Methicillin-resistant Staphylococcus aureus. ** Chi-square test.

## Data Availability

The data will be sent to applicants after their request to the corresponding author. The candidates must agree to a data access contract.

## Abbreviations

The following abbreviations are used in this manuscript:

VAP	Ventilator-associated pneumonia
PICU	Pediatric intensive care unit
MDR	Multidrug-resistant
MRSA	Methicillin-resistant *Staphylococcus aureus*
ESBLs	Extended-spectrum β-lactamases
DLA assay	Double-Layer Agar
PFU	Plaque-forming unit
TEM	Transmission Electron Microscopy
NHSN	National Healthcare Safety Network
